# Cervical prolapse in pregnancy

**DOI:** 10.11604/pamj.2022.42.253.35247

**Published:** 2022-08-05

**Authors:** Aarshika Singh, Ashirwad Sankhe

**Affiliations:** 1 Department of Obstetrics and Gynaecology, Jawaharlal Nehru Medical College, Wardha, Maharashtra, India,; 2 Department of General Surgery, Jawaharlal Nehru Medical College, Wardha, Maharashtra, India

**Keywords:** Cervical prolapse, prolapse, grand multipara, anaemia, pregnancy

## Image in medicine

Cervical prolapse during pregnancy is extremely rare but can cause complications in antepartum or intrapartum period and can have long term sequelae-for mother and fetus. A 32-year-old grand multipara (previous 4 vaginal deliveries) with moderate anaemia (Hb-7.6gm/dl) and poor nutritional status (BMI-17kg/m^2^), presented with 35 weeks´ pregnancy and preterm labor pains with a prolapsed hyperemic cervix 4-5cm dilated protruding out of introitus. We proceeded with fluid and blood products´ resuscitation and close monitoring of labor till delivery of 2100gm male baby with APGAR score 8. Active management of third stage of labor was done with oxytocics to prevent post-partum haemorrhage. After manually repositing the cervix and uterus inside for the time being, the woman was sent to post-natal ward. Daily dressing of prolapse with magnesium sulphate done. A supportive ring pessary with ideal fit for the lady was placed against symphysis pubis resting in the posterior fornix on post-partum day 8. We counseled the lady for interval sterilisation to prevent future pregnancies and telephonic follow-up was taken. Here, multiple vaginal deliveries, anaemia, poor nutritional status were significant contributing factors in both preterm labour pains and cervical prolapse. These etiological factors contribute to major health hazards in developing nations. Surveillance and correction of these comorbidities is of utmost importance in pre-conceptional and antepartum period itself.

**Figure 1:**
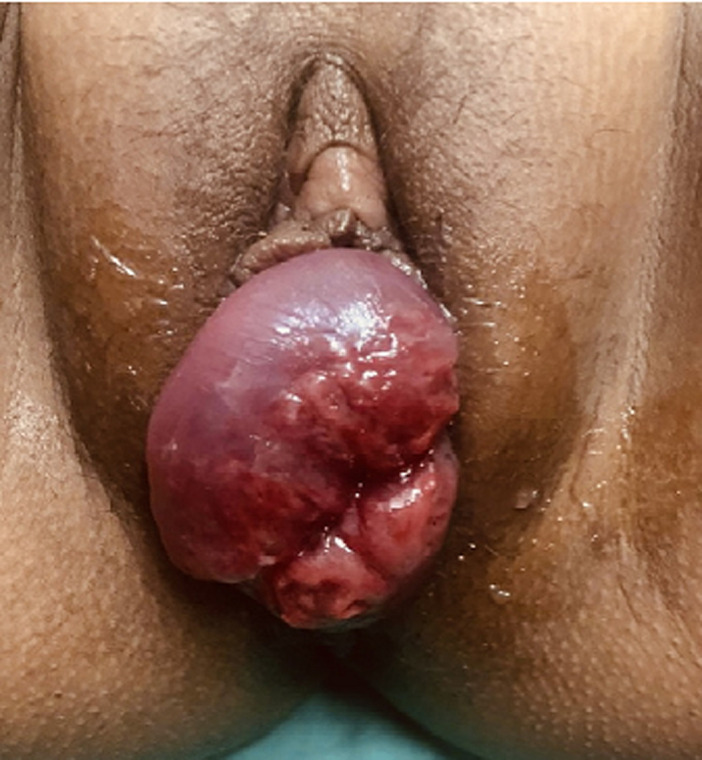
cervical prolapse in pregnancy

